# Biomineral crystallographic preferred orientation in Solenogastres molluscs (Aplacophora) is controlled by organic templating

**DOI:** 10.1038/s41598-024-57754-z

**Published:** 2024-05-05

**Authors:** J. D. Castro-Claros, X. Yin, C. Salas, E. Griesshaber, S. Hörl, A. G. Checa, W. W. Schmahl

**Affiliations:** 1 Departamento de Estratigrafía y Paleontología, Universidad de Granada, 18071 Granada, Spain; 2 Bruker Beijing Scientific Technology, Minhang District, Shanghai, 200233 China; 3 Department of Geo- and Environmental Sciences, Ludwig Maximillians University Munich, 80333 Munich, Germany; 4 Departamento de Biología Animal, Facultad de Ciencias, Universidad de Málaga, 29071 Málaga, Spain; 5 Instituto Andaluz de Ciencias de La Tierra, CSIC-Universidad de Granada, 18100 Armilla, Spain

**Keywords:** Electron microscopy, Marine biology, Structural biology

## Abstract

Aplacophoran molluscs are shell-less and have a worm-like body which is covered by biomineralized sclerites. We investigated sclerite crystallography and the sclerite mosaic of the Solenogastres species *Dorymenia sarsii*, *Anamenia gorgonophila*, and *Simrothiella margaritacea* with electron-backscattered-diffraction (EBSD), laser-confocal-microscopy and FE-SEM imaging. The soft tissue of the molluscs is covered by spicule-shaped, aragonitic sclerites. These are sub-parallel to the soft body of the organism. We find, for all three species, that individual sclerites are untwinned aragonite single crystals. For individual sclerites, aragonite c-axis is parallel to the morphological, long axis of the sclerite. Aragonite a- and b-axes are perpendicular to sclerite aragonite c-axis. For the scleritomes of the investigated species we find different sclerite and aragonite crystal arrangement patterns. For the *A. gorgonophila* scleritome, sclerite assembly is disordered such that sclerites with their morphological, long axis (always the aragonite c-axis) are pointing in many different directions, being, more or less, tangential to cuticle surface. For *D. sarsii,* the sclerite axes (equal to aragonite c-axes) show a stronger tendency to parallel arrangement, while for *S. margaritacea,* sclerite and aragonite organization is strongly structured into sequential rows of orthogonally alternating sclerite directions. The different arrangements are well reflected in the structured orientational distributions of aragonite a-, b-, c-axes across the EBSD-mapped parts of the scleritomes. We discuss that morphological and crystallographic preferred orientation (texture) is not generated by competitive growth selection (the crystals are not in contact), but is determined by templating on organic matter of the sclerite-secreting epithelial cells and associated papillae.

## Introduction

The Aplacophora form a clade of mollucs that consists of the classes Solenogastres and Caudofoveata. Up to now, the Aplacophora diversified to about 440 different species^1,2^. The Solenogastres consist of about 300 and the Caudofoveata of about 140 species, respectively^2–11^.

Aplacophora molluscs are small-sized organisms; their size varies from very few mm to a few cm^11^. They are fully marine and live from sublittoral to abyssal environments, down to more than 7000 m water depths^7–9,11^. Even though some species are found in shallow water habitats, most representatives of the Aplacophora dwell primarily in the deep sea. Aplacophora molluscs are either benthic (Caudofoveata) or infaunal (Solenogastres).

The Solenogastres form elongate bodies, developed a rudimentary foot, and glide along substrate surfaces. They are often found in association with cnidarian and alcyonacean corals, where they climb and coil around hard skeletal elements^1,2^. Scheltema^12^ suggested that the worm-like shape of the Solenogastres is a derived characteristic, as body elongation is the result of adaptation to an epizoic life style. In addition, the worm-like body shape is combined with a marked reduction of the foot. According to Scheltema^12^, the two latter characteristics initiate that the Solenogastres body is, in cross-section, almost circular.

The Solenogastres body and organs are covered by a coat of mineralized skeletal elements, the scleritome, formed of sclerites. Solenogastres sclerites consist of aragonite and have spicular morphologies. The sclerites are hollow, and have, however, strongly mineralized, thick, sclerite walls^1,2,5,7,11^.

Aplacophora molluscs are much studied (see Ponder et al.^11^ and references therein). However, most of these concentrate on morphological, developmental, ecological and evolutionary aspects of the molluscs. The mineralized envelope of the Aplacophora, the scleritome, remained, up to now, little investigated. The studies of^13–15^ form exceptions. However, Haas & Claus^13^ and Ivanov and Scheltema^14^ describe only morphological aspects of Aplacophora sclerites and deduce their results mainly from light microscopy and some SEM imaging. The authors show, for Caudofoveata molluscs that these have flattened sclerites and suggest that morphology, length and width of the sclerites vary for species of the different Aplacophora groups, families and genera^13,14,16^. In a recent study Wendt et al.^15^ use high-resolution SEM, TEM and AFM imaging techniques to highlight, for the Caudofoveata species *Falcidens* sp., sclerite surface, architecture and sub-micrometer scale internal structure.

In the study presented here we discuss the crystallography of Solenogastres sclerite aragonite. We focus on three Solenogastres species and present crystallographic aspects of the aragonite of individual sclerites, and deduce the mode of sclerite aragonite organization in the scleritome. EBSD is a highly suited analytical technique for the measurement of crystallographic axes orientations of crystals. Accordingly, with EBSD we obtain the microstructure and texture of the aragonite of individual sclerites and, when measured for a multitude of crystals in a structural hard tissue, also for the scleritome. We complement crystal orientation results with FE-SEM and laser confocal microscopy imaging for overviews of sclerite morphology, geometry and arrangement pattern in the scleritome.

We choose to investigate the sclerites of the Solenogastres molluscs *Dorymenia sarsii* (Koren and Danielssen, 1877), *Anamenia gorgonophila* (Kowalevsky, 1880) and *Simrothiella margaritacea* (Koren and Danielssen, 1877). We describe the, for a Solenogastres species, prevailing arrangement of aragonite crystallites and:

1. highlight differences in aragonite crystal organization for the assemblies of sclerites in the scleritomes.
2. Illustrate and discuss differences and similarities in sclerite microstructure and texture.
3. Address aragonite texture formation and discuss, for the investigated Solenogastres species, whether the texture is generated by physical determinants, e.g. the type of the crystal growth process, or if the texture is controlled by biological determinants, e.g. characteristics of epithelial cells, located on the surface of the papillae.

## Results

**Figure 1:**
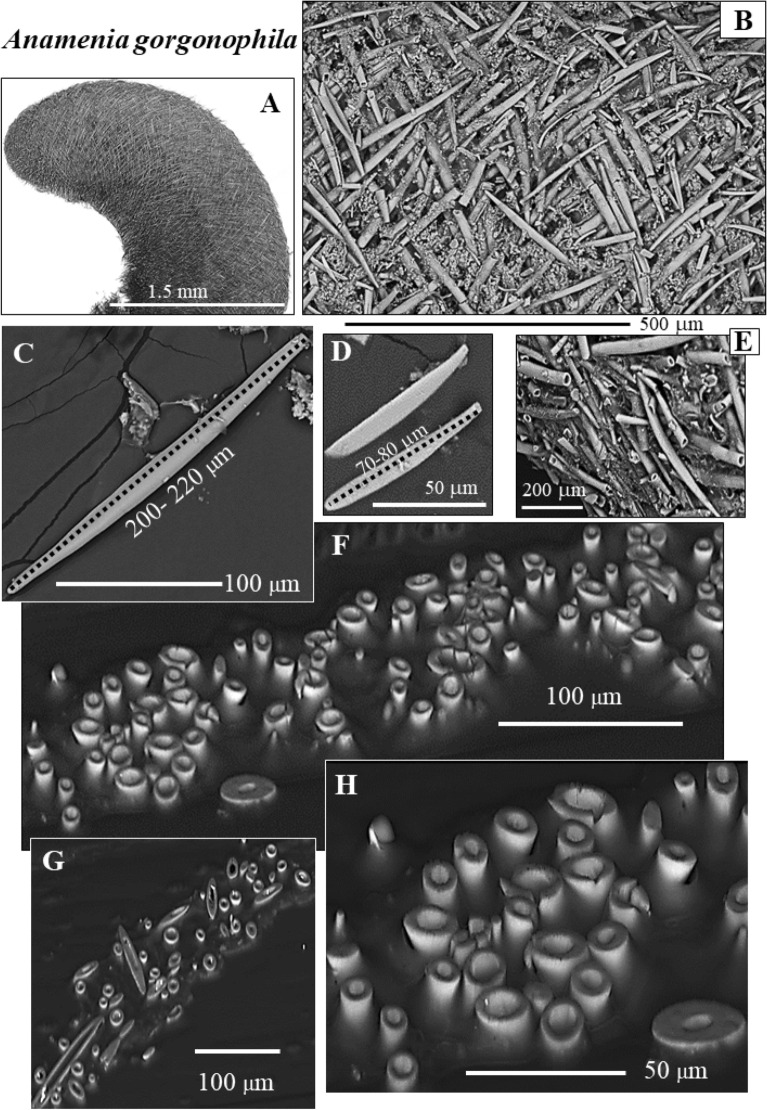
BSE micrographs of the sclerite envelope and of individual sclerites that surround the cuticle and soft tissue of the Solenogastres mollusc species *A. gorgonophila* (**A**–**H**). We observe a rather random arrangement of the sclerites in the scleritome (**A**, **B**). We find long sclerites (**C**, sclerite length exceeding 200 µm) and short sclerites (**D**, sclerite length below 100 µm). The sclerites of *A. gorgonophila* are hollow (**E**–**H**). sclerite wall thickness is uniform (**F**–**H**) and varies between 2 and 3 µm.

**Figure 2:**
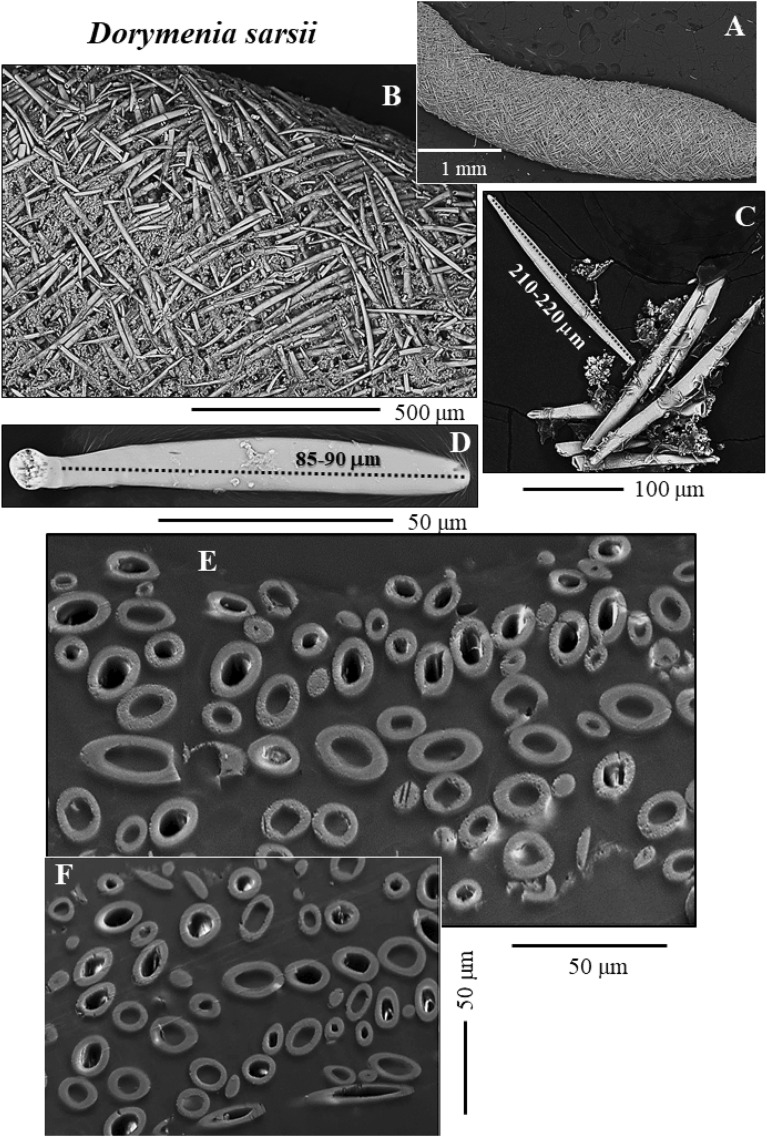
The sclerites that surround the cuticle and soft tissue of the Solenogastres mollusc species *D. sarsii* (**A**–**F**). (**A**–**F**) BSE micrographs of the sclerite envelope and of individual spicules. Based on their length, the sclerites can be grouped into two groups: short sclerites with lengths up to 80–100 µm (**D**) and long sclerites with lengths exceeding 200 µm (**C**). The sclerites of *D. sarsii* are hollow (**B**, **E**, **F**). Sclerite wall thickness is in the range of a few micrometers and is rather uniform (**E**, **F**).

**Figure 3:**
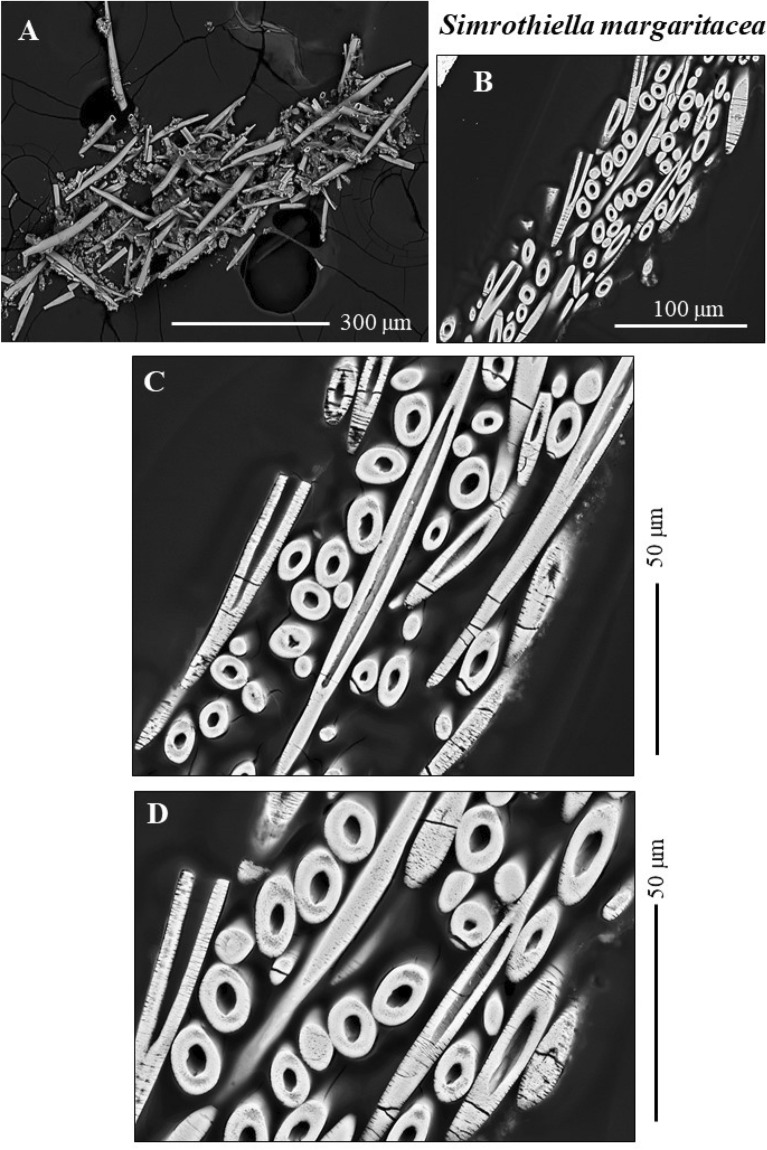
BSE (**A**) and SE (**B**, **C, D**) micrographs of sclerites that envelope the cuticle of the Solenogastres mollusc *S. margaritacea*. The sclerites are hollow. Their maximal length is about 220–240 μm.

**Figure 4:**
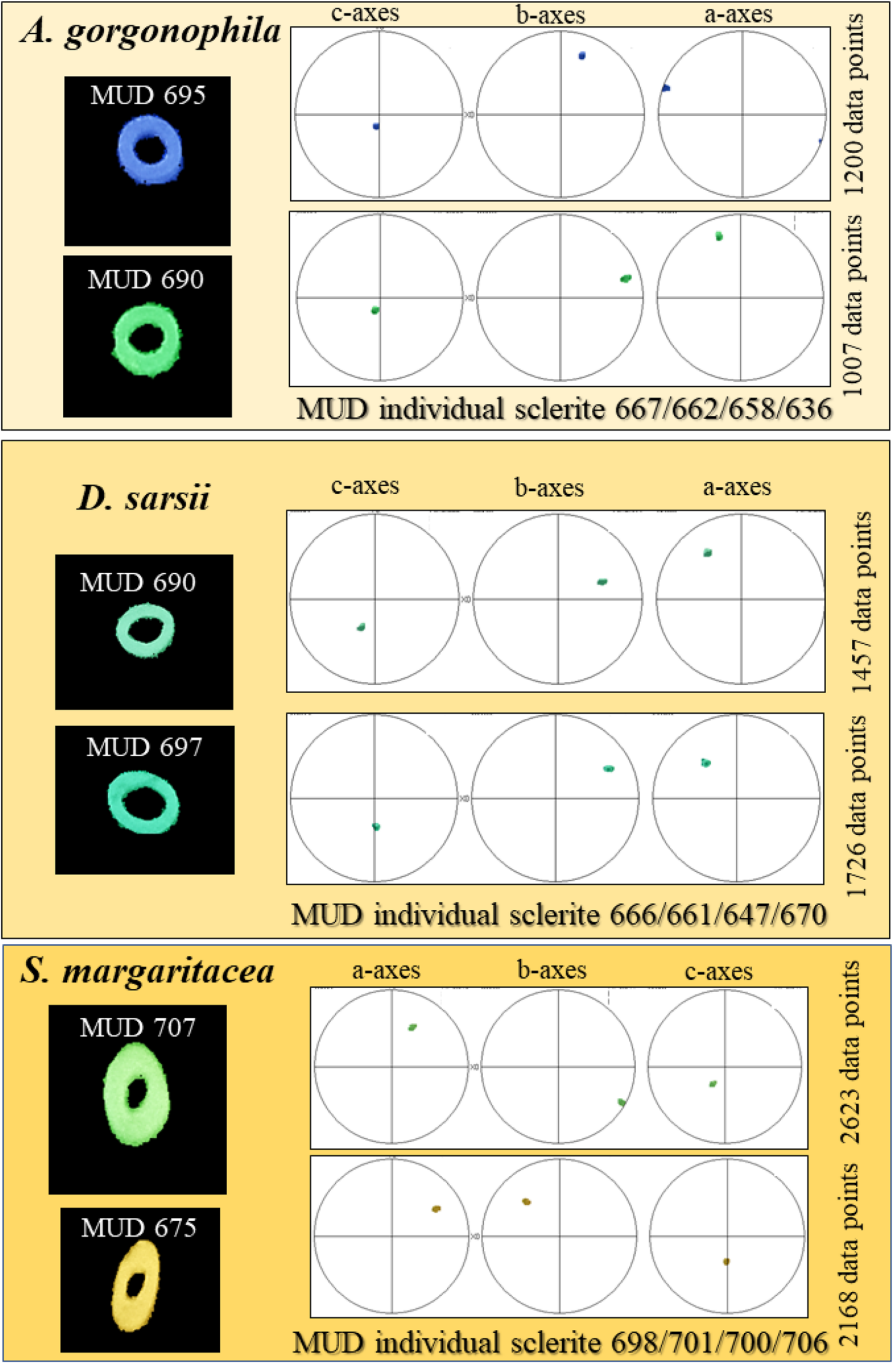
The strongly increased co-orientation strength of aragonite in *A. gorgonophila*, *D. sarsii*, and *S. margaritacea* individual sclerites. We show, for individual sclerites (given on the left), aragonite crystallographic axes orientation with pole figures. Each pole figure shows several hundred crystal orientation data points (figures given on the right-hand side of the pole figure). As these have similar orientation, the data points fall on top of each other. *MUD* multiple of uniform distribution. MUD values are very close to or, even, slightly above 700 and indicate the single-crystallinity of an individual sclerite.

**Figure 5:**
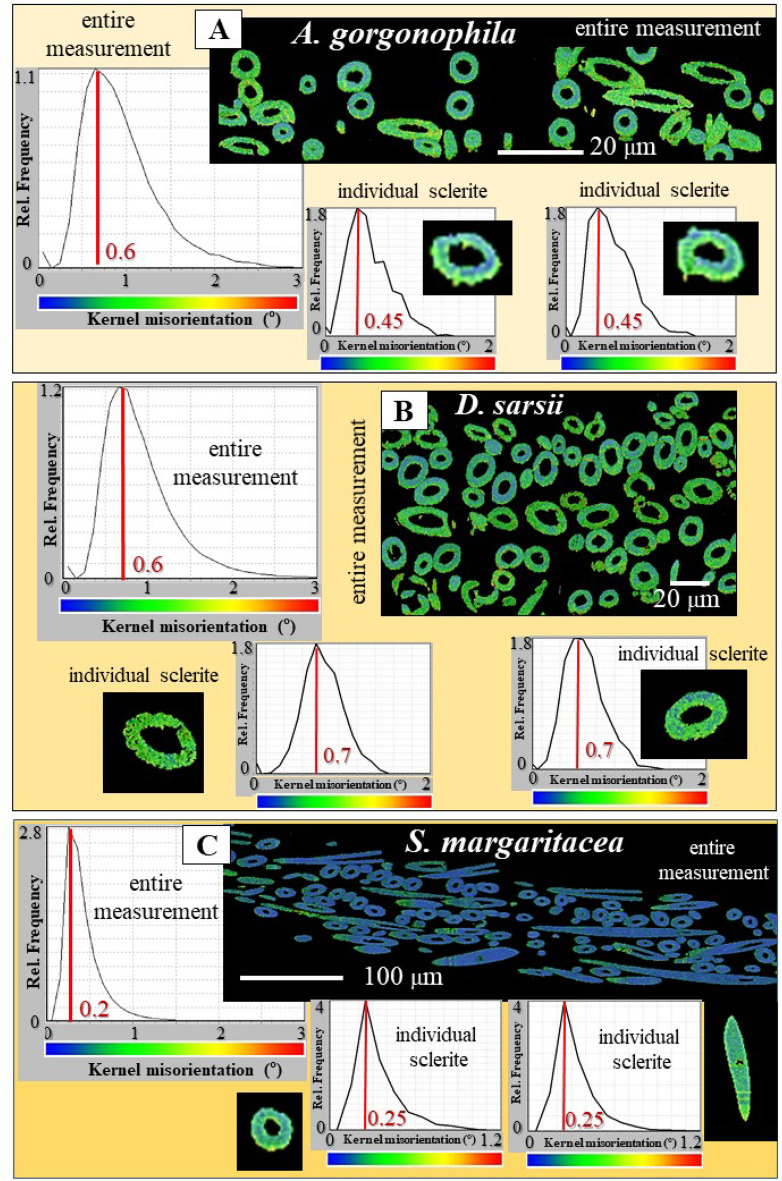
Aragonite co-orientation strength in the scleritome and in individual sclerites of *A. gorgonophila*, *D. sarsii* and *S. margaritacea* (**A**–**C**). We present color-coded Kernel misorientation maps, calculated from EBSD measurements. These give, in color, the degree of misorientation between neighboring crystals; the used color-code is given below the corresponding relative frequency—misorientation diagram. Maximal misorientation for portions of the scleritome (for an entire EBSD scans) ranges up to 3°. For individual sclerites maximal misorientation scatters between 1° and 2°. Most co-oriented are aragonite crystallites in the sclerites of *S. margaritacea* (**C**). Slightly less co-oriented is the aragonite that comprises the sclerites of *A. gorgonophila* (**A**) and *D. sarsii* (**B**). At maximal frequency, the misorientation values for individual sclerites are 0.45° and 0.7° for *A. gorgonophila* and *D. sarsii*, respectively. For *S. margaritacea*, at maximal misorientation, the misorientation between crystallites is as low as 0.2°–0.25°.

**Figure 6:**
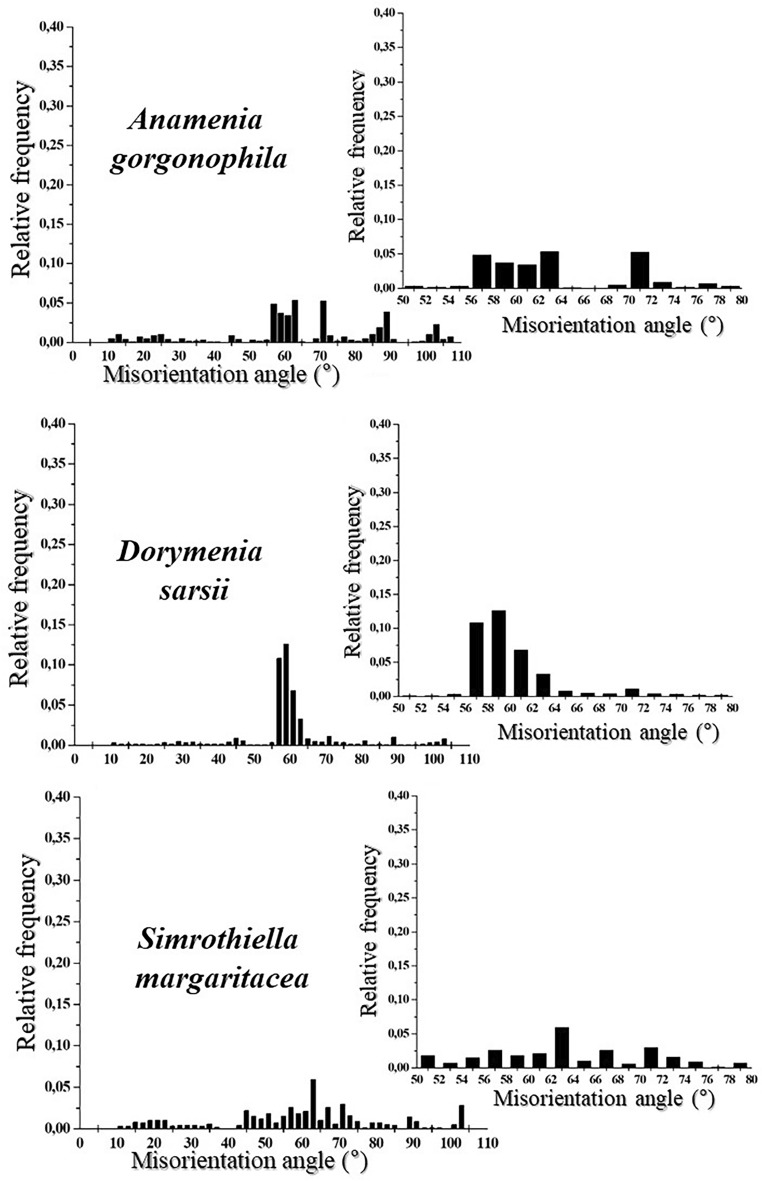
The aragonite that forms the sclerites of *A. gorgonophila*, *D. sarsii* and *S. margaritacea* is not twinned. Relative frequency—misorientation angle diagrams for the three investigated Solenogastres molluscs. We observe a wide range in aragonite misorientation. Relative frequency—misorientation angle diagrams of further EBSD scans is shown in Fig. S2.

**Figure 7:**
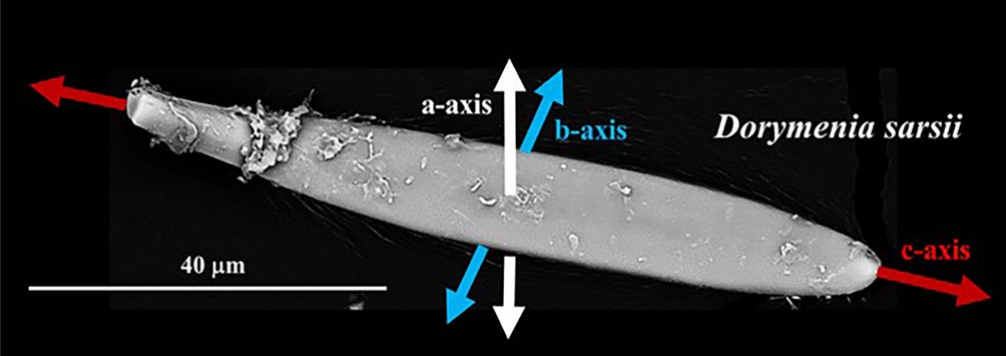
The orientation of aragonite a-, b-, c-axes in a sclerite of *D. sarsii*. For the other investigated Solenogastres species we find similar aragonite axes orientations, relative to the morphological, long axis of the sclerite. Aragonite c-axis is parallel, while aragonite a-axis and b-axis are within the diameter of the sclerite.

**Figure 8:**
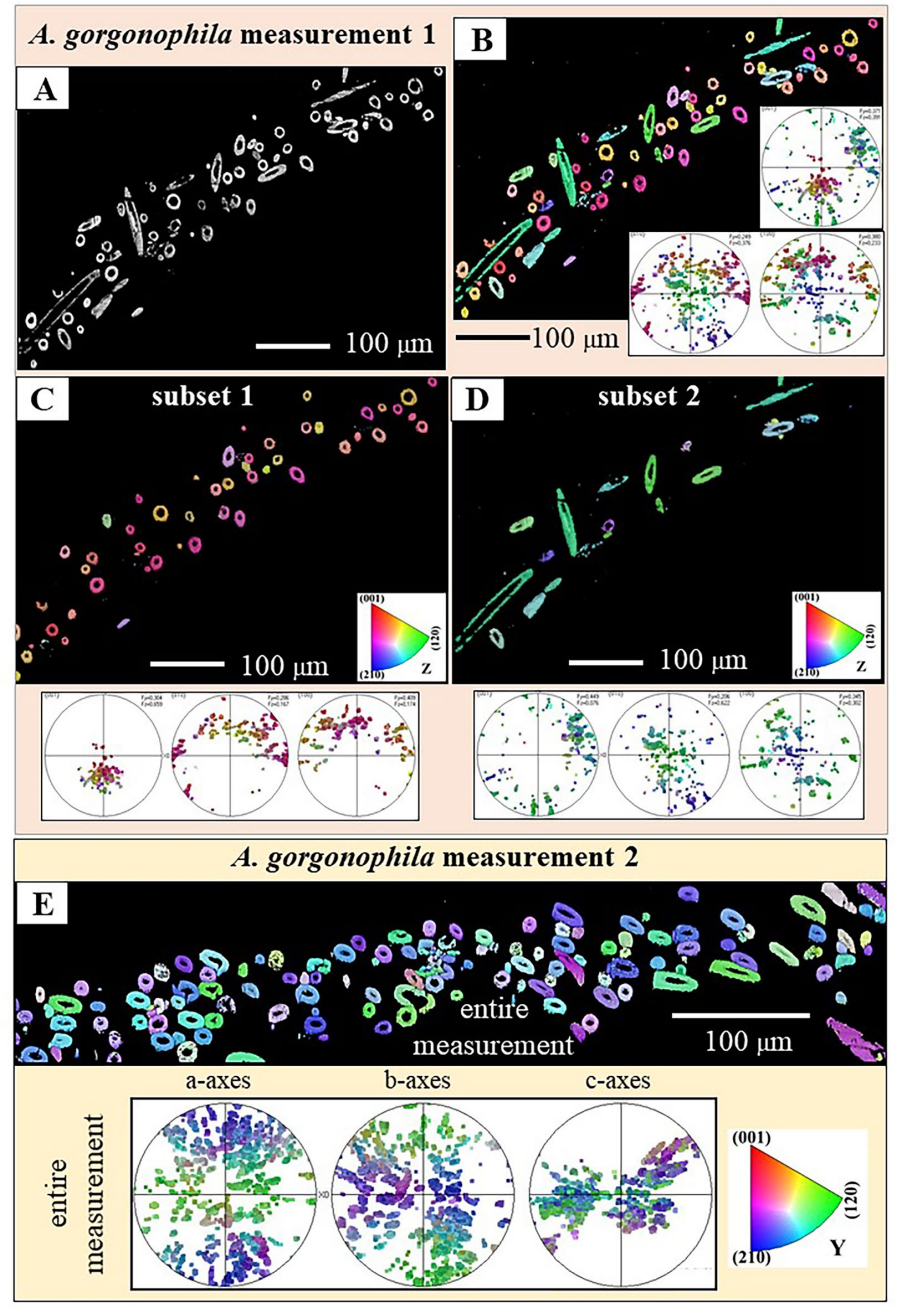
Pattern of sclerite and aragonite crystal orientation for the scleritome of *A. gorgonophila*, shown with color-coded EBSD maps and corresponding pole figures (**A**–**E**). We show results of two EBSD-scans (**A**–**D**, and **E**). Sclerite morphological, long axis orientation is little structured (**A**). Accordingly, aragonite c-axis orientation is also little structured and cannot be assigned to, e.g. different layers of sclerites with a specific sclerite orientation, or some other arrangement pattern. According to our results, sclerite and aragonite crystal organization within the scleritome of *A. gorgonophila* is significantly less organized, as it is the case for scleritome of *D. sarsii* (Fig. 9) or that of *S. margaritacea* (Fig. 10).

**Figure 9:**
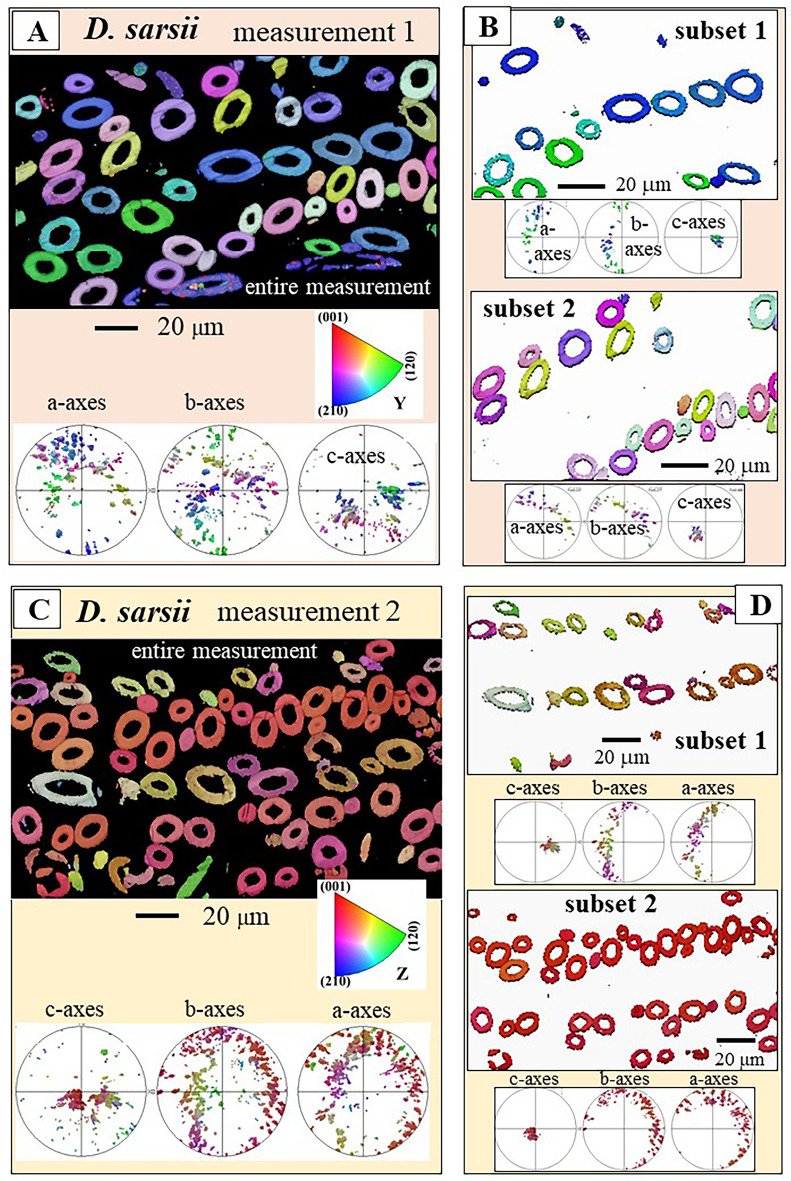
Pattern of sclerite arrangement and crystal orientation organization within the scleritome of *D. sarsii*. We show two EBSD scans (**A**, **C**); crystal orientation variation is given with color-coded EBSD maps and corresponding pole figures (**A**–**D**). We observe for the sclerites that form the scleritome of *D. sarsii,* c-axes clusters. See pole figures in (**A**–**D**). These clusters can be assigned to sets of alternating layers of sclerites; see EBSD maps and pole figures in (**B**, **D**). Accordingly, sclerite arrangement in the scleritome of *D. sarsii* is structured.

**Figure 10:**
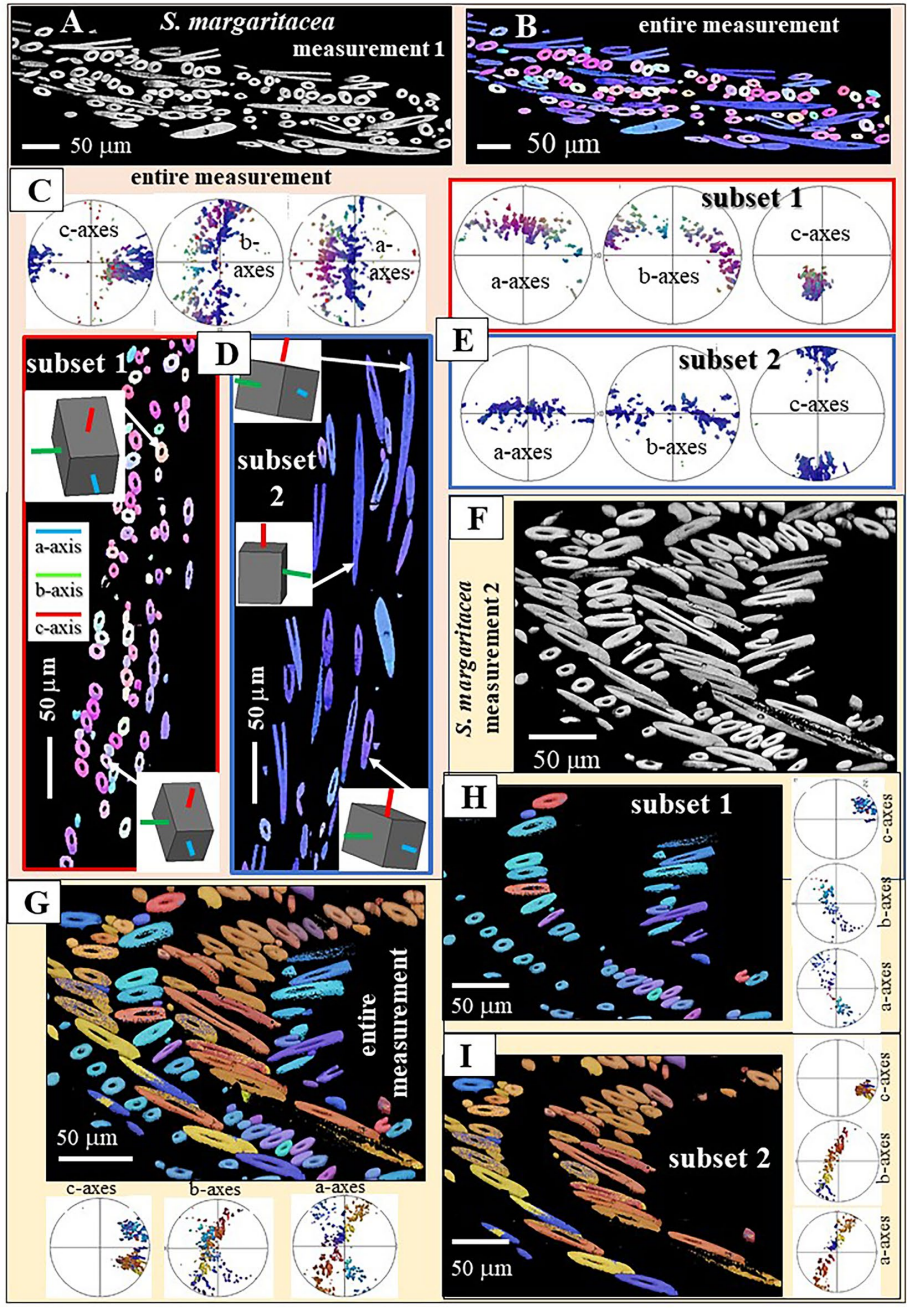
Sclerite arrangement and mode of aragonite crystal orientation in the scleritome of *S. margaritacea*. Of the three investigated Solenogastres species, most structured is the aragonite and sclerite organization in the scleritome of *S. margaritacea*. We show results of two EBSD scans, crystal orientation data with color-coded EBSD maps and corresponding pole figures (**A**–**I**). About five rows of sclerites surround the cuticle and soft tissue of *S. margaritacea*. Sclerite morphological, long axis orientation alternates between adjacent layers of sclerites (**A-D**, **E**–**I**). For one layer morphological, long axis orientation is within the plane of view (**D**, subset 2), for the other layer morphological, long axis orientation is perpendicular to the plain of view (**D**, subset 1). As c-axis orientation is always parallel to the morphological, long axis orientation of the sclerite, we observe in the pole figures well-defined c-axes clusters (see pole figures in **C**, **E**, **G**, **H**, **I**). Aragonite c-axis orientation is within (e.g.  **E**, subset 2) or/and, more or less, perpendicular to the plane of view (e.g. **E**, subset 1). Sketched crystals in **D** show crystal orientation for individual sclerites. (**A**, **F**) EBSD band contrast measurement map; (**B**, **D**, **G**, **H**, **I**) EBSD crystal orientation map.

The investigated Solenogastres molluscs construct the mineralized cover that surrounds their cuticle and organs of a meshwork of sclerites (Figs. 1, 2, 3, 4, 5, 6, 7, 8, 9, 10, S1, S2). The sclerites of the investigated Solenogastres species have spicular morphologies, are hollow and, with exception of the sclerites that encase the soft body of *S. margaritacea*, show a low to decreased structural organization. In particular, negligible structural organization is observed for the sclerites of *A. gorgonophila*.

As shown for *A. gorgonophila* (Fig. 1), *D. sarsii* (Fig. 2), *S. margaritacea* (Fig. 3), when based on individual sclerite length, the sclerites can be subdivided into two groups: (1) short sclerites with lengths between 80 and 100 µm and (2) long sclerites reaching lengths up to 220–230 µm (Figs. 1, 2, 3). Individual sclerites are single crystals (Fig. 4). To the knowledge of the authors, such a strong crystal co-orientation strength has not yet been reported for other carbonate biological hard tissues, not even for sea urchin shells and spines. The calcite of the latter was, so far, regarded to be single-crystalline. EBSD measurements allow the determination of the degree of co-, or/and misorientation between crystals^17,18^. A crystallized material is a single crystal when all its crystals are fully co-aligned. In this case, crystallographic axes orientations for all crystals in the EBSD map are on similar spots in the corresponding pole figure and the MUD value (see Terminology in Methods) of the EBSD map is very close to 700, or even above. As for biocarbonate hard tissues this is a very important finding, we demonstrate sclerite single-crystallinity with crystal orientation data for individual sclerites in pole figures (Fig. 4) as well as MUD values calculated for individual sclerites.

Figure 4 shows, for each of the three investigated species, EBSD scans of two individual sclerites. For all the scans there is almost no scatter for the a-, b- and c-axes orientations (Fig. 4), although, each pole figure gives a few hundred crystal orientation data points. Accordingly, irrespective of the investigated species, aragonite crystallites that comprise individual sclerites are strongly co-oriented. Most co-oriented are sclerite crystallites of *S. margaritacea*. MUD values for the latter are 700 and above, the maximal relative frequency value in the Kernel misorientation diagram is as low as 0.25° (Figs. 4, 5). Hence, for most of the crystallites that comprise *S. margaritacea* sclerites, misorientation between neighboring crystallites is only about 0.25°. Slightly less co-oriented are aragonite crystalites in the sclerites of *D. sarsii* and *A. gorgonophila*. MUD values for individual sclerites, for the latter two species, scatter between 640 and 670 (Fig. 4). In the Kernel misorientation diagram the maximal relative frequency value is 0.45°–0.6° for *A. gorgonophila* and 0.7° for *D. sarsii*, respectively (Fig. 5). Hence, for the majority of crystallites in *D. sarsii* and *A. gorgonophila* sclerites, misorientation between adjacent crystallites is not higher than 0.6°–0.7°. However, it is not as low as 0.25°, as it is the case for the sclerites of *S. margaritacea*. The difference in Kernel misorientation for crystal orientation between *S. margaritacea*, on the one hand, and *A. gorgonophila* and *D. sarsii*, on the other, is small, it is, however, significant. Kernel misorientation was determined for all EBSD scans performed in this study. Determination of the MUD value was done for several sclerites of the investigated Solenogastres species.

The phenomenon of crystal twinning is an important characteristic for the material in question and is often observed for biologically secreted aragonite. If crystals are twinned, or not, can unequivocally be deduced from EBSD measurements. All our measurements show that the aragonite of the investigated Solenogastres sclerites is not twinned (Figs. 6, S2). This is in contrast to the aragonite that forms the skeletal elements (spicules, scales, plates) of the Polyplacophora^18^ as well as the shells of many other molluscs. Lack of aragonite twinning is given with the single-crystallinity of individual sclerites. Nonetheless, as the relative frequency—misorientation angle diagrams show (Figs. 6, S2), we find for the aragonite of the investigated Solenogastres species a wide range in crystal misorientation (1) misorientation between crystallites up to 110°, as well as (2) clusters in misorientation at about 70–72 and 55–61° (Figs. 6, S2). Note that these peaks reflect orientational relations between the aragonite of different spicules, while each spicule is an untwinned single crystal.

From EBSD measurements made on individual sclerites we can deduce crystallographic axes orientation of aragonite crystallites (Fig. 7). This is important for understanding the mode of crystal orientation within the entire scleritome. We find that for all investigated Solenogastres species each individual sclerite has the c-axis of the aragonite (the aragonite lattice) parallel to the morphological long axis of the sclerite. As aragonite is orthorhombic, the a- and b-axes are perpendicular to the morphological, long axis of the sclerite (Fig. 7). This crystallographic axis orientation pattern is valid for the sclerites of all investigated Solenogastres species.

Figures 8, 9 and 10 show the microstructure and texture of the scleritomes of the investigated Solenogastres species. EBSD maps visualize sclerite organization (the microstructure), pole figures (deduced from EBSD maps) give the mode of aragonite crystal orientation (the texture) of the scleritomes. We show and discuss two EBSD scans per Solenogastres species. We show always the entire scans as well as subsets (parts) of these (Figs. 8, 9, 10).

For *A. gorgonophila* we find a very low degree of structural arrangement of aragonite crystals within the scleritome (Fig. 8). This is due to the very little structured mode of sclerite orientation in the scleritome (Fig. 8B). Nonetheless, the sclerite arrangement in the scleritome is not entirely random (Fig. 8B) and, accordingly, we find clusters of aragonite c-axis orientation in the pole figures of the mapped sections of the scleritome (Fig. 8B-D). In essence, sclerite organization and mode of c-axis orientation for *A. gorgonophila* are significantly less clear-cut, in comparison to that in the scleritome of *D. sarsii* and, especially, in the scleritome of *S. margaritacea*.

The sclerite envelope that covers the soft tissue of *A. gorgonophila* consists of sclerite assemblies where neither aragonite crystallite, nor sclerite morphological axis orientation is structured. This contrasts with what is observed for the scleritome of *D. sarsii* (Fig. 9). For the latter we find per EBSD scan two aragonite c-axes clusters (see the pole figures in Fig. 9A, C). Each of these can be assigned to a set of rows of sclerites (see subset 1 and subset 2 EBSD maps in Fig. 9B, D). Hence, when based on crystal orientation, the scleritome of *D. sarsii* consists of alternating sets of sclerite rows and we find for these different modes of aragonite orientation (see pole figures for subsets 1 and 2 in Fig. 9B, D).

The sclerite arrangement that surrounds the soft tissue of *S. margaritacea* is outstanding (Fig. 10). It is the most structured sclerite arrangement that we find for the three investigated Solenogastres species. When based on sclerite/aragonite crystallite orientation, we observe for the sclerite envelope of *S. margaritacea* rows of differently oriented sclerites (Fig. 10A, B, D, G, H, I). Within one particular row of sclerites the morphological axis of the sclerites is perpendicular to the plane of view, while in the adjacent row sclerites, sclerite morphological axis orientation is within the plane of view (Fig. 10). As aragonite c-axis orientation is always parallel to the morphological, long axis of the sclerite (Fig. 7), the pronounced difference in sclerite morphological axis orientation implicates that in the pole figures we observe two different and clear-cut patterns of crystal orientations (Fig. 10C, E, G, H, I).

## Discussion

### The sclerites

The Solenogastres molluscs that were investigated in this study surround their soft tissue with a 150–200 µm thick cover of spicule-shaped mineralized elements, the sclerites. In cross-section we find 4 to 6 rows of sclerites that encase the soft body of the molluscs (Figs. 1, 2, 3, S1).

The sclerites are separate mineralized entities. Adjacent sclerites are not connected and only rarely touch each other (Figs. 1F–H, 2E, F). The sclerites are secreted by epithelial cells of the cuticle^19,20^ and are associated with epithelial papillae^20–24^. Beedham and Trueman^24^ showed, that at full development of the sclerite, the contact between papillae and the fully secreted sclerite is formed by an organic cap. The latter is firmly attached to the base of the sclerite and to the papillae^24^. Recent ultrastructural results of Castro-Claros et al.^19^ demonstrate that Solenogastres sclerites originate in cell membrane invaginations. The authors show as well that individual epithelial cells secrete only one sclerite. This contrasts with the secretion of shells of some other marine organisms. It has been shown, for example, for brachiopods, that at secretion of a brachiopod fiber, five to eight neighboring cells need to communicate and to interact^25^.

MUD values and Kernel misorientation evaluations (Figs. 4, 5) demonstrate that the aragonite crystallites of individual sclerites are not (for the sclerites of *S. margaritacea*), or only slightly (for the sclerites of *A. gorgonophila* and *D. sarsii*), misoriented to each other. Individual sclerites are single-crystals. Accordingly, separate epithelial cells secrete separate aragonite single-crystals, each cell a single-crystalline sclerite. This is of main importance and is shown with the work of Castro-Claros et al.^19^ and this study. Accordingly, for Solenogastres sclerites epithelial cells determine:

1. the pattern of aragonite a-, b-, c-axis orientation for the sclerite they secrete (Fig. 7),
2. the morphology and dimension of individual sclerites (Figs. 1, 2, 3),
3. single-crystallinity of individual sclerites (Figs. 4, 5),
4. lack of twin formation within individual sclerites (Figs. 6, S2)
5. the mode of sclerite arrangement in the scleritome (Figs. 1, 2, 3, 11, 12).

**Figure 11:**
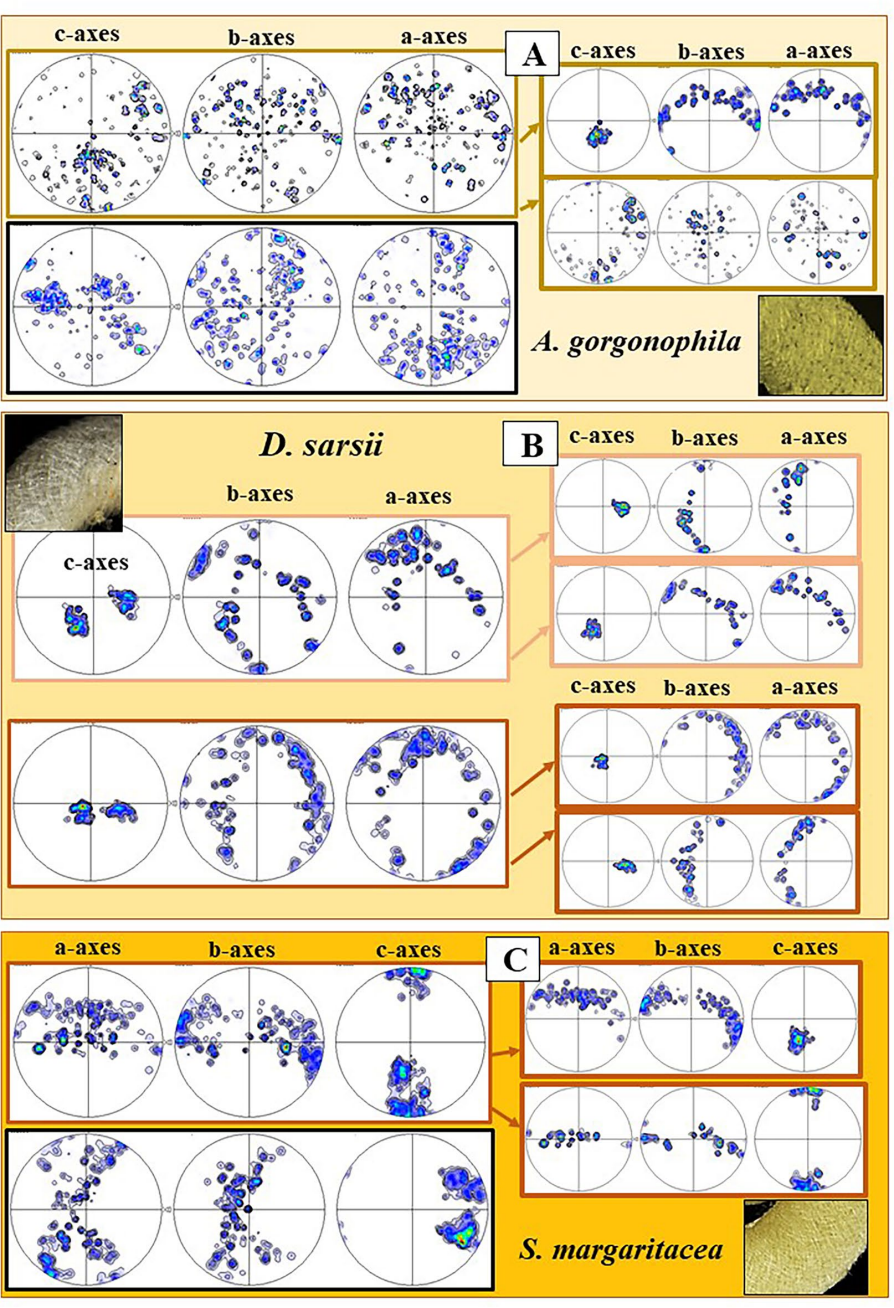
Summary of aragonite texture patterns for the sclerites in the scleritome of *A. gorgonophila* (**A**), *D. sarsii* (**B**) and *S. margaritacea* (**C**). (**A**–**C**) Crystal orientation data shown as density distributions, complemented with a laser confocal microscopy image depicting sclerite organization.

**Figure 12:**
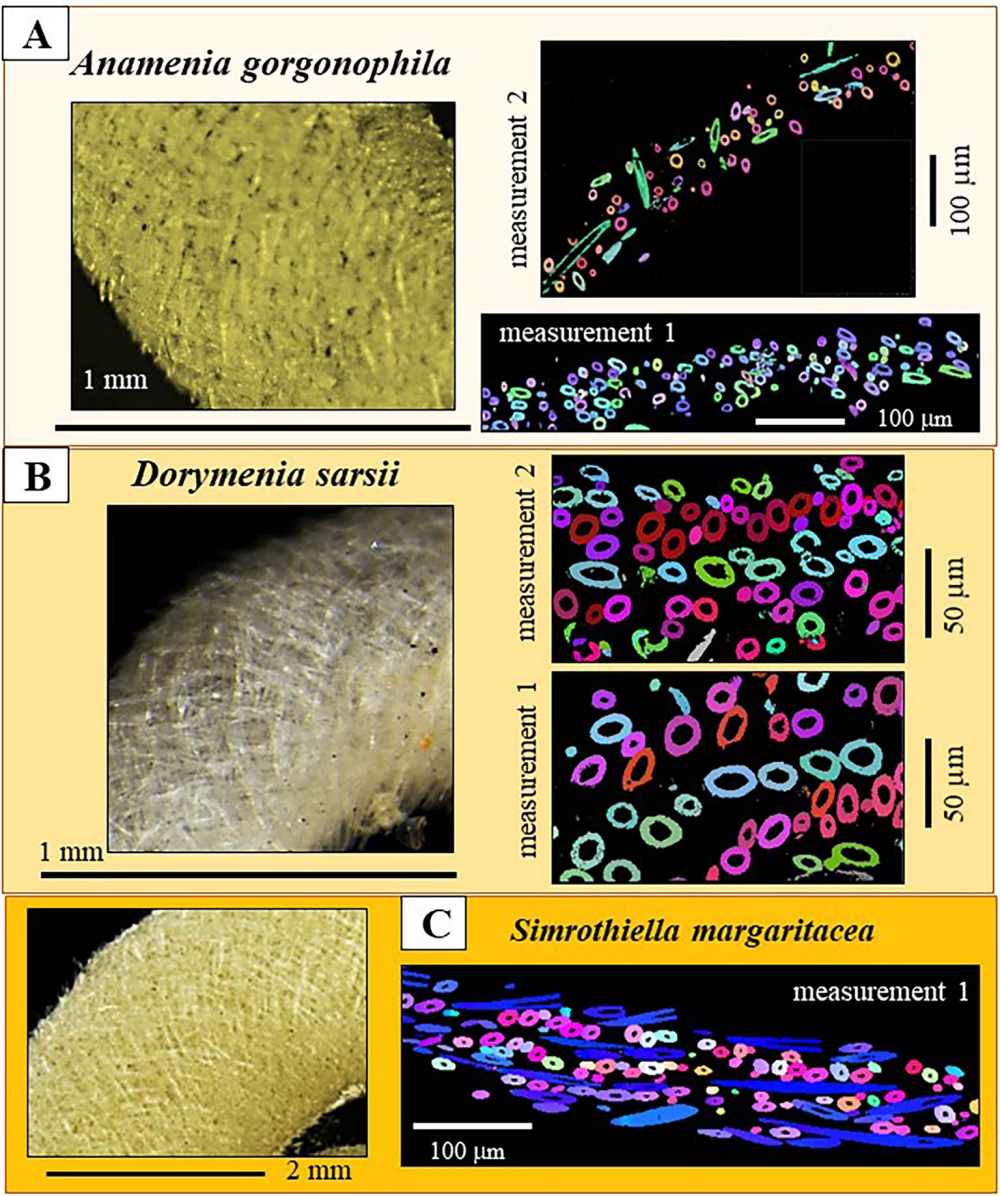
Summary of sclerite organization patterns in the scleritome of *A. gorgonophila* (**A**), *D. sarsii* (**B**) and *S. margaritacea* (**C**). (**A**–**C**) Laser confocal microscopy image depicting some structural characteristics of sclerite organization (left) together with EBSD maps (right) visualizing the mode of sclerite organization in the scleritome.

Hence, the composition of biopolymers secreted by epithelial cells and ultrastructural characteristics of epithelial papilla and cuticle cells, determines all structural and microstructural characteristics of sclerite aragonite. None of the above-mentioned crystallographic and structural/microstructural characteristics of sclerite and scleritome aragonite are induced by purely physical parameters and controls, e.g. the anisotropy of the growth speed of the aragonite crystals or the growth mode of crystals, but only by biological determinants, such as cellular, physiological and biopolymer characteristics of the sclerite-secreting epithelial cells and their association to the papillae of the cuticle. This contrasts to the formation of bivalve and brachiopod microstructures, where both physical processes as well as combinations of physical and biological controls are determinants of crystal morphology, dimension and organization^25–27^.

The single-crystallinity of individual sclerites is outstanding. So far, such a high crystal co-orientation strength as that observed for, e.g. the sclerites of *S. margaritacea*, has not been found for other biological carbonate hard tissues. Even the calcite of sea urchins (tests or spines) comes only close to the single-crystallinity of *S. margaritacea* aragonite^28,29^. Sea urchin calcite appears to be single-crystalline on the macroscopic scale. However, on the nanometer scale sea urchin calcite exhibits an extended defect and dislocation network. This accounts for the mosaicity^28,29^ of the sea urchin calcite and is the reason why it is not entirely single-crystalline. MUD values of sea urchin calcite scatter between 630 < MUD < 650 and do not reach an MUD of 700. For the sclerites of the investigated Solenogastres species we find often values close to an MUD of 690 to 700, or even above an MUD of 700.

### The microstructure of the scleritomes

Solenogastres molluscs have vermiform body shapes and live on substrate surfaces. They have a rudimentary foot and are able to glide on uneven surfaces or/and to climb and curl around the hard parts of branching organisms^1,2^. Hence, the scleritome of Solenogastres molluscs does not have to be only protective but also flexible and light-weight. This is, indeed, what we find. Protection by the scleritome is achieved with formation of a mineral cover consisting of some rows of interlaced, stiff spicules. Individual spicules are fracture-resistant, as they have strongly mineralized walls. Flexibility is given by the interlaced arrangement of the sclerites which are not fused to each other. The sclerite cover of the investigated Solenogastres species is a 3D crosslink of spicular mineralized skeletal elements. The structure of the scleritome resembles the structure of a woven fabric. The light-weight character of the scleritome is called forth by the hollowness of the spicules; as, as little heavy mineral material as possible is used.

It is most surprising to find that Solenogastres molluscs vary the mode of crystal organization in their scleritome (Figs. 11, 12). The *A. gorgonophila* scleritome is almost unstructured (Fig. 12A), the pattern of aragonite crystal orientation within the tangential plane of the cuticle is close to random (Fig. 11A). For the *D. sarsii* scleritome we observe formation of rows of sclerites (Fig. 12B). Adjacent rows have slightly different sclerite orientations (Fig. 11B). Accordingly, for *D. sarsii*, we find in the pole figures quite distinct clusters of aragonite a-, b-, c-axes orientations for the different, but adjacent, sclerite layers (Fig. 11B). Most structured is the aragonite of the scleritome of *S. margaritacea* (Figs. 11C, 12C). This mollusc species varies sclerite orientation systematically in the scleritome and forms adjacent layers with, more or less, orthogonally oriented sclerites. Accordingly, as aragonite c-axis is always parallel to the morphological, long axis of the sclerite, we find, for *S. margaritacea*, clear-cut clusters and patterns for a-, b, -c-axes orientations in the pole figures (Fig. 11C).

### The textures of individual sclerites and of the scleritome

Based on crystal orientation measurements, we can identify for Solenogastres sclerite and scleritome aragonite three different texture patterns. We find for individual sclerites of all three investigated mollusc species a single-crystal texture. We observe for *D. sarsii* and *S. margaritacea*, for aragonite crystal and sclerite assembly within the scleritome, an axial texture, and for aragonite crystal and sclerite assembly in the scleritome of *A. gorgonophila,* a disordered texture. Even though for *A. gorgonophila* the texture of individual sclerites is single-crystalline, as the orientation pattern of sclerites in the scleritome is almost random, the texture pattern of aragonite within the scleritome of *A. gorgonophila* is also almost random. It is worth noting that, for sclerite assemblies, textures refer to the arrangement of individual crystals (single-crystal spicules) and not, as usual, to a crystalline aggregate.

What initiates these different texture patterns?

For shelled organisms (conchiferan molluscs, brachiopods, cephalopods) the main microstructure and texture determinants are assessed by now^25–27^. These are:

A. *Growth of crystals by growth competition*; e.g. formation of argonauta prismatic calcite^30,31^, brachiopod columnar calcite^32^, bivalve myostracal aragonite^33,34^, and rotaliid foraminifera calcite^35,36^.
B. *Growth of crystals between biopolymer membranes that form through self-organization* prior to mineralization; e.g. formation of interlamellar membranes of molluscan nacreous aragonite^37–40^.
C. Generation of microstructure and texture through the *interaction of crystal surfaces with biopolymers of an extended fibrous extracellular matrix*; e.g. formation of mytilid bivalve fibrous calcite^41^.
D. *Influence and effect of mantle epithelial cells that, at secretion, are in contact with the composite hard tissue*; e.g. formation of bivalve columnar prisms and brachiopod fibrous and primary layer calcite^25,26,32^.

Crystallographic preferred orientation, or texture, can develop during the nucleation step of crystal formation. Preferred orientation, or texture, is epitaxial if the nucleation becomes oriented on a template. Furthermore, preferred orientation, or texture, can develop during growth of crystals influenced/controlled by growth competition, or it can develop by strong plastic deformation.

Our EBSD results show that individual sclerites are single-crystals. SEM images show that adjacent sclerites are not in contact, each sclerite is a separate crystal entity, a separate single crystal (Figs. 1, 2, 8, 9, 10, S1). Extracellular organic matrices, as observed for the shells of bivalves, gastropods, cephalopods and brachiopods do not occur in the scleritomes of the investigated Solenogastres molluscs. Hence,

1. The texture patterns that we find for the sclerites and scleritome of the investigated Solenogastres species cannot be explained by the influence of growth competition on crystal orientation (A), as the crystals do not touch each other.
2. Individual sclerites are sheathed by an organic envelope^18^, however, this forms simultaneously with the growing sclerite aragonite in the cell membrane invaginations^19^. Hence, the mode of Solenogastres sclerite formation is different from the mode of secretion described in (B). Furthermore, scenario (B) does not lead to crystallographic preferred orientation per se.
3. Scenario (C) does not apply also, as the sclerites are not in contact and do not form a compact mineral/organic composite. An extended extracellular biopolymer matrix joining the crystals is absent in the scleritome of the investigated Solenogastres species.
4. Crystallization in the epithelial cell invaginations^19^ bears some relationship with the scenario described in (D) but again, this scenario does not explain formation of crystallographic texture, the preferred orientation of sclerite aragonite, per se.

The sclerites of the investigated mollusc species are solely attached to epithelial cells and papillae of the cuticle, at distances of a few tens of nanometers. Hence, the different textures that we measure for Solenogastres sclerite aragonite can only be induced by specific ultrastructural characteristics of epithelial, papilla and cuticle cells and their invaginations. The aragonite of Solenogastres spicules and the calcite of rotaliid foraminifera^35,36^ are the only examples so far where it can be shown that crystallographic axes orientation of the biocrystals is already determined at the onset of the mineral formation process, at nucleation, and that it is solely influenced by organic substance and the associated biopolymers.

## Conclusions

Aplacophora molluscs cover their body with a mosaic of biomineralized skeletal elements, sclerites. For the investigated Solenogastres species the sclerites are spicule-shaped and have specific arrangement patterns in the scleritome. The Solenogastres scleritome is a highly evolved hard tissue that renders protection of the soft body from external threats at concomitant retention of flexibility for movement and climbing of the organism on uneven surfaces. The conjunction of these properties is enabled by (1) the specific structural characteristics of individual sclerites and (2) the mode of sclerite arrangement in the scleritome.

In this study we highlight and discuss aragonite crystal orientation of individual sclerites as well as aragonite crystal and sclerite organization in the scleritome. For the Solenogastres species *Dorymenia sarsii*, *Anamenia gorgonophila*, and *Simrothiella margaritacea* we draw the following conclusions:

1. In cross-section about 5–6 layers/rows of sclerites surround the soft tissue of the investigated Solenogastres mollusc species.
2. Solenogastres sclerites are spicule-shaped, hollow and have densely-mineralized spicule walls.
3. Crystallographic axes characteristics, aragonite lattice orientation, of individual sclerites are similar for all three investigated Solenogastres species.
4. Aragonite a-, b-, c-axes orientation, the orientation of the aragonite crystal lattice within a sclerite, is such that aragonite c-axis is parallel to the morphological, long axis of the sclerite. Consequently, aragonite a-, and b-axes are perpendicular to the morphological, long axis of the sclerite.
5. The aragonite within individual sclerites is strongly co-oriented and is not twinned.
6. Individual sclerites can be regarded to be single-crystals.
7. In contrast to inorganic aragonite crystals, which grow at their tip, Aplacophora sclerite aragonite grows at the base. Hence, growth polarity is inverted for Aplacophora aragonite, relative to inorganic aragonite.
8. The spicules are arranged to the soft tissue with a criss-cross arrangement pattern, resembling a woven fabric.
9. For the investigated Solenogastres mollusc species we find different sclerite organization patterns in the respective scleritome. Sclerite arrangement in the *A. gorgonophila* scleritome is almost random in all tangential directions to the cuticle. Sclerite arrangement in the *D. sarsii* scleritome is more structured. Most structured is the assembly of sclerites in the scleritome of *S. margaritacea*. The sclerites in the latter form rows and have, in these, orthogonal orientations.
10. For all three investigated Solenogastres species, crystal orientation in individual sclerites, the single-crystallinity of sclerites, the untwinned nature of sclerite aragonite, the organization pattern of sclerites in the scleritome, the microstructure as well as the texture of the scleritome is controlled by epithelial cells, papillae and associated biopolymers.

## Materials and methods

### Materials

We investigated the sclerites of the Solenogastres species *Dorymenia sarsii* (Koren and Danielssen, 1877), *Anamenia gorgonophila* (Kowalevsky, 1880), and *Simrothiella margaritacea* (Koren and Danielssen, 1877) (Table 1). We investigated per species three specimens. One specimen was imaged with laser confocal microscopy and FE-SEM imaging techniques; two specimens were scanned with EBSD. EBSD measurements were performed on various parts of the scleritome.

**Table 1:** The investigated Solenogastres species, their sampling locations, the conducted expedition and date of sampling.

Species	Class	Family	Sampling location	Expedition sampling date
Anamenia gorgonophila	Solenogastres	Strophomeniidae	35° 57.64′ N–02° 52.54′ W 35° 58.89′ N–02° 53.98′ W	Expedition CIRCAESAL 0721 05/08/2021
Dorymenia sarsii	Solenogastres	Proneomeniidae	36° 26.508′ N–7° 00.0.85′ W 36° 26.031′ N-7° 00.120′ W	Expedition INTEMARES A4 CAD 13/04/2021
Simrothiella margaritacea	Solenogastres	Simrothiellidae	Galicia Bank	Expedition INTEMARES BANGAL 2011

The taxonomy of the studied species follows WoRMS Editorial Board (2024). World Register of Marine Species. Available from https://www.marinespecies.org at VLIZ. Accessed 2024-01-27. doi:10.14284/.

### Methods

#### Light microscopy and FE-SEM imaging

Prior to EBSD measurements samples were imaged with a Keyence 3D laser scanning microscope (VK-X1000 series) and an FE-SEM (Hitachi SU5000).

#### Electron backscattered diffraction (EBSD) measurements

Aplacophoran species were embedded either into a very fluid EPON resin or into superglue. Embedded/glued samples were trimmed, cut and polished in an ultramicrotome with trimming, glass and diamond knives.

For EBSD measurements the samples were coated with 4–6 nm of carbon. Measurements were carried out on a Hitachi SU5000 field emission SEM, equipped with an Oxford Instruments Nordlys II EBSD detector. At measurement, the SEM was operated at 15, 18 or/and 20 kV, as it was necessary for obtaining high-quality crystal orientation data. Data were collected and evaluated using Oxford Instruments AZtec and CHANNEL 5 HKL software. EBSD measurements were performed with step increments of 200 nm. For each species we investigated with EBSD two specimens. Each specimen was scanned with, at least, 4 maps, for some species, up to 8 EBSD maps. Individual measurements lasted between 10 and 12 h. EBSD scans were performed on different parts of the scleritome.

#### Terminology

We use, in this contribution, the terms microstructure and texture in a crystallographic, material science, sense. Crystallographic axes orientation results were gained from electron backscatter diffraction (EBSD) measurements^42^. EBSD is a fully automated microdiffraction method that provides space-resolved information on the phase state of the material and on crystallite orientation with a precision in the order of 0.15°–0.2°^42^. The spatial resolution depends on the system and the sample in question, it is currently in the order between 100 and 200 nm for carbonates and 100 nm or better for metals^42^. For more detailed information concerning the EBSD technique see^42^.

The *microstructure* of a crystalline material is given by the arrangement pattern of crystallographic axes orientation of crystals. Hence, the microstructure of a structural material is the assemblage of 3D orientations of crystal lattices of the constituting crystals^42^. In this study, *microstructures* are presented with grey-scaled EBSD band contrast measurement maps as well as with colour-coded EBSD orientation maps. The used colouring code is indicated in the figure or stated in the figure caption.

In the color-coded crystal orientation maps, similar or different colours indicate similar or different crystallite orientations^42^. EBSD band contrast measurement images depict the backscattered signal strength in each measurement point. A high signal strength corresponds to light grey colours and indicates strong diffraction at the crystal lattice. Dark colours are indicative of non-diffracting substances, e.g. polymers, or of an overlap of minute crystallites that cannot be resolved (indexed) automatically with the EBSD acquisition software^42^.

The *texture* of a crystalline material is the nature of crystallographic lattice and crystal orientation^42^. The texture of a material is the mode of crystallographic preferred orientation of crystals^42^. In this study, the *texture* is presented with pole figures that give either the measured orientation data or the density distributions of these. For the density distributions, we use the lowest possible setting for half width and cluster size in the CHANNEL 5 software: a half width of five and a cluster size of three degrees. The half width controls the extent of the spread of the poles over the surface of the projection sphere, a cluster comprises data with the same orientation^42^.

An *axial texture* is given when the c-axes show co-orientation (clustering in the pole figure around a single direction), while the corresponding a- and b-axes vary in orientation on a great circle perpendicular to the texture axis, in this case, the c-axis direction^42^.

From crystal orientation measurements we can calculate crystal *co-orientation strength*. The latter is given with MUD values which are derived from density distributions of the measured crystal orientation data. The MUD (multiple of uniform -random- distribution) value is calculated with the Oxford Instruments CHANNEL 5 EBSD software. A high MUD indicates high crystal co-orientation strength, while low MUD values reflect low to negligiable strength of crystallite or/and mineral unit co-orientation. With a half width of five and a cluster size of three degrees, an MUD value of 1 indicates random orientation distribution and no preferred orientation, an MUD value or higher than 700 documents almost perfect crystallite co-orientation, a single-crystal-like co-orientation of crystallites^17,42,43^.

We process data gained from EBSD measurements for the visualization of *crystal misorientation patterns*: local Kernel misorientation and crystal misorientation with respect to a chosen reference orientation. *Local Kernel misorientation* shows the deviation in orientation between neighboring measurement points, in this study, calculated for 3 × 3 clusters. Misorientation results are given color-coded, the used color-code is given with the figure. Deviation in orientation corresponds to internal strain, e.g. caused by incorporation of biopolymers^17,42,43^.

We show relative frequency—misorientation angle diagrams. Data were calculated with the CHANNEL 5 software from EBSD scans. We observe a multitude of misorientations; these scatter between 5° and 100°^42^.

We use in this contribution the terms competitive growth and self organization.

When *crystallization takes place through competitive growth*, many crystals nucleate close to each other in random orientations, and, at growth, compete for space. As crystal growth speed is anisotropic, the growth development of crystals is orientation-selective. The result of the growth competition process is a strong decrease in the number of crystals as one moves away from the nucleation substrate, accompanied by an increase in crystal size and generation of a progressively stronger crystal co-orientation strength.

*Self-organization* is connected to liquid crystallization and is a process where overall order forms from interactions between parts of an initially less ordered or even disordered system.

### Supplementary Information

Supplementary Information 1.

## Data Availability

The datasets generated during the current study are available from the corresponding author on request.
