# Supplementary material for: Biomineral crystallographic preferred orientation in Solenogastres molluscs (Aplacophora) is controlled by organic templating
Source: Sci Rep. 2024 May 5;14:10309. doi: 10.1038/s41598-024-57754-z (PMC11070423; doi:10.1038/s41598-024-57754-z)
Supplement: Supplementary file 1 — Supplementary Information 1. [file 41598_2024_57754_MOESM1_ESM.docx]

**Biomineral crystallographic preferred orientation in Solenogastres molluscs (Aplacophora) is controlled by organic templating**

J. D. Castro-Claros^1^, X. Yin^2, 3^, C. Salas^4^, E. Griesshaber^3,*^, S. Hörl^3^, A. G. Checa^1,5^, W. W. Schmahl^3^

1. Departamento de Estratigrafía y Paleontología, Universidad de Granada, 18071 Granada, Spain

2. Bruker, Beijing, Scientific Technology, Minhang District, Shanghai 200233, China

3. Department of Geo- and Environmental Sciences, Ludwig Maximillians University Munich, 80333 Munich, Germany

4. Departamento de Biología Animal, Facultad de Ciencias, Universidad de [Málaga](https://scholar.google.es/citations?view_op=view_org&hl=es&org=9410994602360350752), 29071 [Málaga](https://scholar.google.es/citations?view_op=view_org&hl=es&org=9410994602360350752), Spain

5. Instituto Andaluz de Ciencias de la Tierra, CSIC-Universidad de Granada, Armilla, 18100, Spain


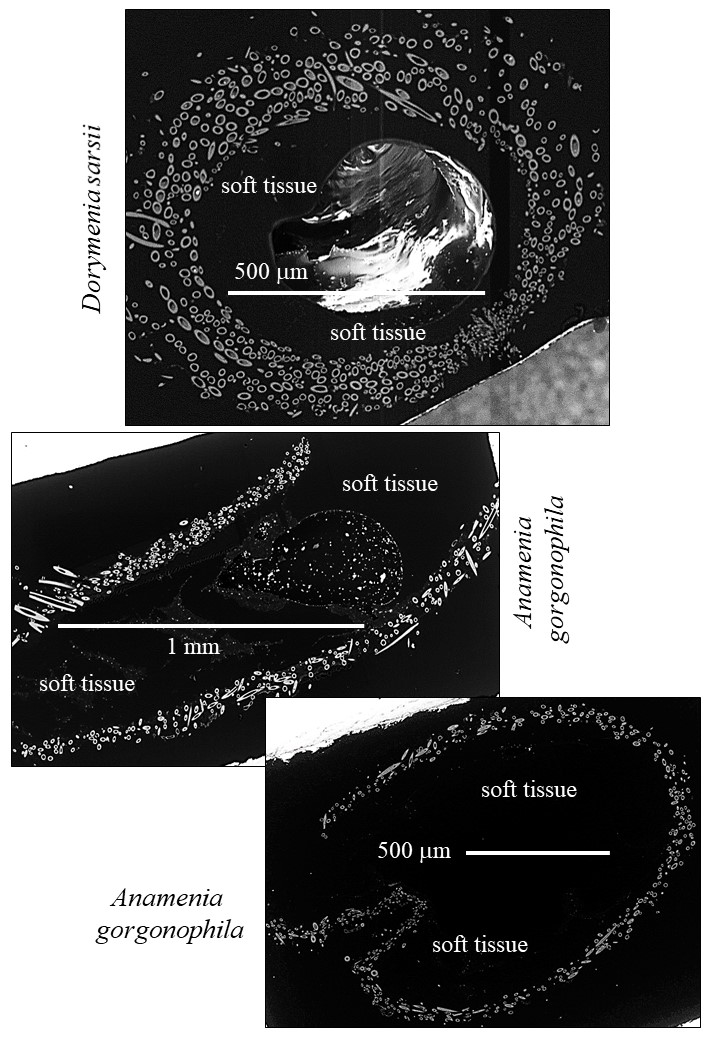


**Figure S1.** BSE micrographs of the sclerite envelope that surrounds the soft tissue and cuticle of the Solenogastres species *D. sarsii* and *A. gorgonophila*. An about 150 to 200 µm thick layer of loosely assembled sclerites comprise the sclerite envelope. Note: The sclerites do not touch each other. The sclerite envelope of *S. margaritacea* is shown in Fig. 8.


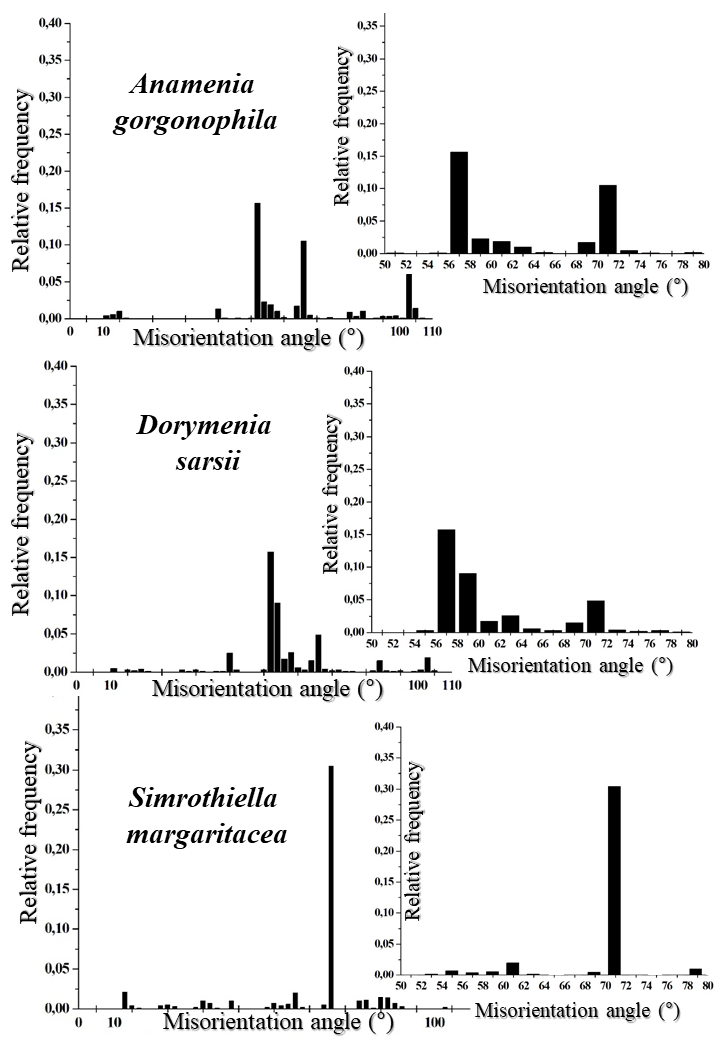


**Figure S2.** Relative frequency versus misorientation angle diagrams for the sclerite envelope of the investigated Solenogastres species. The diagram on the right-hand side is a zoom-in into the diagram on the left-hand side. We observe a wide range in misorientations.
